
JOURNAL INFORMATION
==============================
NLM Title Abbreviation: Behav Neurol
Journal ID: Behav Neurol
NLM Journal ID: 8914585
Journal ID: BN

Behavioural Neurology

ISSN: 0953-4180
EISSN: 1875-8584
Publisher: Wiley

ARTICLE INFORMATION
==============================
PMCID: PMC5434383
PMID: 21422563
DOI: 10.3233/BEN-2010-0303
Article ID: 148485
Article version: 1
Subjects: Research Article

Neuropsychological Evidence of High-Level Processing in Binocular Rivalry

Daini Roberta 1 *
Facchin Alessio 1
Bignotti Marco 1
Lentini Cristina 1
Peverelli Milena 2
O'Shea Robert P. 3
Molteni Franco 2
1 Psychology Department University of Milano-Bicocca Milan Italy
2 Centro di Riabilitazione “Villa Beretta” Costamasnaga (LC) Italy
3 Psychology Southern Cross University Coffs Harbour Australia
*Roberta Daini: roberta.daini@unimib.it
Print publication date: 2010
Electronic publication date: 2011 Mar 7
Volume: 23
Issue: 4
First page: 233
Last page: 235
Received 2011 Jan 31; Accepted 2011 Jan 31
Copyright: Copyright © 2010 Hindawi Publishing Corporation and the authors.
Copyright year: 2010
License: This is an open access article distributed under the Creative Commons Attribution License, which permits unrestricted use, distribution, and reproduction in any medium, provided the original work is properly cited.
License URL: https://creativecommons.org/licenses/by/3.0/

==============================
Behavioural Neurology 23 (2010) 233–235
DOI 10.3233/BEN-2010-0303
IOS Press

### Neuropsychological evidence of high-level processing in binocular rivalry

Roberta Daini[a,*], Alessio Facchin[a], Marco Bignotti[a], Cristina Lentini[a], Milena Peverelli[b], Robert P. O'Shea[c] and Franco Molteni[b]
[a]*Psychology Department, University of Milano-Bicocca, Milan, Italy*
[b]*Centro di Riabilitazione "Villa Beretta", Costamasnaga (LC), Italy*
[c]*Psychology, Southern Cross University, Coffs Harbour, Australia*

One of the most fascinating and challenging questions of cognitive neurosciences is what are the neural correlates of consciousness. We try to answer this question by studying patients having a dissociation between conscious perception and sensory stimulation.

We studied patients with right brain damage (RBD) involving unilateral spatial neglect (USN). USN follows a lesion in the right frontoparietal cortex and involves lack of consciousness of visual stimuli (or parts of them) presented in the contralesional (left) side of space, without any sensory deficit [5]. Even if patients are unable to describe the left side of a stimulus, they show behavioural evidence of preserved visual analysis when tested implicitly [4].

To study visual consciousness in neglect patients, we used binocular rivalry. This involves showing different images to the two eyes. Instead of seeing both images simultaneously, neurologically normal people see one image for a few moments with no trace of the other, then they see the other image for a few moments with no trace of the first, then they see the first again, and so on for as long as they care to look. This phenomenon exhibits a dissociation between sensory input, which is unchanging, and consciousness, which changes continuously.

There are two opposing explanations of binocular rivalry: low-level and high-level theories [8]. Blake [2] proposed that rivalry is resolved at a low level of the visual system, in the primary visual cortex, involving inhibitory interactions between monocular representations of the two rival stimuli. Logothetis et al. [6], on the other hand, proposed that rivalry is resolved at a high level of the visual system, in the inferotemporal cortex or higher, involving interactions between representations of the two rival images.

In support of high-level theories, neuroimaging studies have consistently shown that binocular rivalry involves activity in the parietal and frontal areas (e.g. [7, 10]), particularly in the right hemisphere. These are the areas that if damaged are associated with USN, so we decided to examine whether binocular rivalry requires these areas to be intact.

There are three possible outcomes:

1. If binocular rivalry is determined by low-level processes, USN patients should show the same rivalry as neurologically healthy subjects.
2. If binocular rivalry is determined by high-level processes, RBD patients should show compromised rivalry.
3. If binocular rivalry depends critically on the areas responsible for USN, such patients should show compromised rivalry that is different from that shown by RBD patients without USN.

After neuropsychological evaluation, we selected two groups of right-handed RBD patients: with unilateral spatial neglect (USN+; 9 patients) and without neglect (USN-; 12 patients). The neuropsychological and demographic data for the two groups are visible in Table 1. We also studied 10 healthy, right-handed, control subjects (CTRL), matched for age ($M = 50.4$;

*Corresponding author. E-mail: roberta.daini@unimib.it.

ISSN 0953-4180/10/$27.50 © 2010 – IOS Press and the authors. All rights reserved

Table 1

Demographic and neurological details of the right brain-damaged patients, with and without unilateral spatial neglect (USN). I = infarct; H = hemorrhage; T = temporal; P = parietal, BG = basal ganglia; IC = internal capsula; O = occipital. MMSE adj = MMSE adjusted score score; *= MMSE raw data

| Group | Name | Sex | Age | Edu. | Month onset | Lesion | Lesion site | VFD | MMSE adj | AlbertL | Albert R | DillerL | DillerR | BellL | BellR | LB 16 | WJL | WJR | flowers | Reading |
|---|---|---|---|---|---|---|---|---|---|---|---|---|---|---|---|---|---|---|---|---|
| USN- | CA | F | 55 | – | – | I | T, P | 0 | 24* | 1 | 0 | 15 | 13 | 5 | 7 | −4.6 | 0 | 0 | 0 | 5 |
| USN- | RS | M | 53 | 8 | 26 | – | – | 0 | 27.97 | 0 | 0 | 0 | 0 | 1 | 0 | 1 | 0 | 0 | 0 | 6 |
| USN- | GR | M | 43 | 13 | 3 | I | F, T, P | 0 | 27.89 | 0 | 0 | n.a. | n.a. | 4 | 0 | 0.4 | 0 | 0 | 0 | 6 |
| USN- | VM | F | 35 | 13 | 24 | I | F, P | 0 | 27.75 | n.a. | n.a. | n.a. | n.a. | 1 | 0 | 1.2 | 0 | 0 | 0 | 6 |
| USN- | CF | M | 69 | 13 | 6 | H | T,P | 0 | 30 | 0 | 0 | 0 | 0 | 0 | 0 | 1.2 | 0 | 0 | 0 | 6 |
| USN- | FC | F | 50 | 13 | 9 | H | F, P, BG | 0 | 30 | 0 | 0 | 7 | 9 | 3 | 0 | −2.8 | 0 | 0 | 0 | 6 |
| USN- | LS | F | 44 | 8 | 14 | H | F, P | 0 | 29.62 | 1 | 0 | 0 | 0 | 5 | 5 | −1 | 0 | 0 | 0 | 6 |
| USN- | ES | M | 71 | 8 | 3 | I | – | 0 | 30 | 0 | 0 | 9 | 8 | 0 | 2 | 0.8 | 2 | 2 | 1 2 | 6 |
| USN- | IM | M | 37 | 14 | 13 | H | – | 0 | 26.1 | 0 | 0 | 1 | 0 | 1 | 3 | −2.4 | 0 | 0 | 1 | 6 |
| USN- | AB | M | 67 | 8 | 2 | H | F, IC | 0 | 27.47 | 0 | 0 | 0 | 2 | 2 | 2 | 1.6 | 0 | 0 | 0 | 6 |
| USN- | AT | F | 63 | 17 | 1 | I | F | 0 | 25.46 | 0 | 0 | 7 | 7 | 4 | 3 | −2 | 1 | 0 | 0 | 6 |
| USN- | MG | F | 67 | 13 | 3 | I | T, P, IC | 0 | 26.46 | 0 | 0 | 5 | 0 | 0 | 4 | 2.8 | 0 | 0 | 0 | 6 |
| USN+ | BT | F | 40 | 13 | 6 | I | – | 0 | 30 | 0 | 0 | 23 | 14 | 12 | 7 | 3 | 1 | 0 | 2 | 4 |
| USN+ | SP | F | 52 | 13 | 1 | I | – | n.a. | 19.99 | 3 | 0 | 53 | 30 | n.a. | n.a. | n.a. | 7 | 0 | 1 2 | 4 |
| USN+ | GB | M | 69 | 5 | 12 | I | T, O, BG | 0 | 23.27 | 5 | 0 | 53 | 29 | 16 | 3 | 31.8 | 16 | 2 | 1 2 | 0 |
| USN+ | SS | M | 40 | 8 | 2 | H | – | 0 | 28.62 | 1 | 0 | 6 | 2 | 5 | 0 | 1 | 0 | 0 | 1 | 6 |
| USN+ | EC | M | 65 | 3 | 1 | I | F, T, P | 0 | 23.99 | 1 | 0 | 53 | 48 | 17 | 14 | −11.2 | 0 | 0 | 0 | 3 |
| USN+ | AA | M | 61 | 13 | 8 | H | F | 0 | 25.49 | 0 | 0 | 5 | 1 | 7 | 0 | 4.4 | 4 | 0 | 1 | 6 |
| USN+ | VM2 | M | 60 | 5 | 4 | I | F, T, P | 0 | 24.27 | 11 | 1 | 53 | 12 | 14 | 3 | 8.8 | 19 | 0 | 1 | 4 |
| USN+ | SC | M | 66 | 13 | 5 | I | T, BG, IC | 0 | 26.49 | 0 | 0 | 8 | 3 | 2 | 0 | 9.6 | 0 | 0 | 1 | 6 |
| USN+ | MG2 | F | 51 | 8 | 10 | I | F, T, P | 0 | 24.97 | 1 | 0 | 17 | 3 | 6 | 2 | 9.8 | 0 | 0 | 1 2 | 6 |

SD = 5.6). All subjects had normal acuity in each eye tested separately and normal binocular vision, defined as simultaneous perception and fusion.

We presented pairs of rival images in a stereoscope that was aligned with the midsagittal plane. Rival images varied in complexity:

– Square-wave gratings tilted 45° clockwise and 45° counterclockwise (Orientation) (simple);
– Square-wave gratings with low and high spatial frequencies (Frequency) (simple);
– Two different configurations of illusory motion (Movement) (moderately complex);
– Pictures of two different objects: a face and a house (Object) (complex).

Stimulus size was between 5.37° and 7.15°.

We asked subjects to press one button on a PC's mouse when they saw one rival stimulus with no trace of the other, and another mouse button when they saw the other rival stimulus with no trace of the first.

Responses were recorded using a laptop PC running a script in ePrime software.

Rival stimuli were presented for 60 seconds, twice to allow counterbalancing of image to eye.

We counted the number of alternations from consciousness of one image to the other. We found that RBD patients reported fewer alternations (M = 3.24) than control subjects (M = 14.81), $F(2, 28) = 25.16$, $p < 0.001$, but with no significant differences between USN+ (M = 4.29) and USN- (M = 2.45) patients. Control subjects also reported more alternations with the orientation stimuli and fewer with the objects than with any other type, whereas RBD patients had similar number of alternations for all stimulus types, $F(6, 84) = 3.19$, $p < 0.01$.

One possible explanation is that RBD patients had a more conservative criterion for deciding on visibility of an image. If so, patients should show not only a reduced number of alternations, but also a reduced number of key presses. However, the ratio between alternations and key presses is much higher for control subjects (0.76) than for RBD patients (0.38), with no significant differences between USN+ and USN-. That is, control subjects' consciousness tended to alternate between the two rival stimuli, whereas RBD patients' consciousness tended to dwell on one of them. This suggests that rivalry is compromised in RBD patients independently of USN (outcome 2).

In conclusion we obtained an effect of stimulus complexity in neurologically normal subjects, confirming a slower rivalry alternation rate for more complex stimuli [1]. Patients with right brain damage did not show this effect and showed a reduced alternation rate. Differently from Bonneh et al. [3] we did not find any differences between patients with and without USN, suggesting that the mechanism underlying the suppression of visual input in binocular rivalry is different from that involved in USN.

Because our patients' brain damage is confined to right parietal and frontal lobes, we can conclude that processing within these areas influences the temporal course of visual consciousness. This is consistent with fMRI [7,10] and electrophysiological [9] studies on neurologically normal subjects, which showed an involvement of the right parietal lobe and the fronto-parietal system in alternation conditions vs stable conditions.

### References

[1] D. Alais and D. Melcher, Strength and coherence of binocular rivalry depends on shared stimulus complexity, *Vision Research* **47**(2) (2007), 269–279.

[2] R. Blake, A neural theory of binocular rivalry, *Psychological Review* **96**(1) (1998), 145–167.

[3] Y.S. Bonneh, M. Pavlovskaya, H. Ring and N. Soroker, Abnormal binocular rivalry in unilateral neglect: evidence for a non-spatial mechanism of extinction, *Neuroreport* **15**(3) (2004), 473–477.

[4] R. Daini, P. Angelelli, G. Antonucci, S.F. Cappa and G. Vallar, Exploring the syndrome of spatial unilateral neglect through an illusion of length, *Experimental Brain Research* **144**(2) (2002), 224–237.

[5] P.W. Halligan, G.R. Fink, J.C. Marshall and G. Vallar, Spatial cognition: evidence from visual neglect, *Trends in Cognitive Sciences* **7** (2003), 125–133.

[6] N.K. Logothetis, D.A. Leopold and D.L. Sheinberg, What is rivalling during binocular rivalry? *Nature* **380** (1996), 621–624.

[7] E.D. Lumer, K.J. Friston and G. Rees, Neural correlates of perceptual rivalry in the human brain, *Science* **280** (1998), 1930–1934.

[8] F. Tong, M. Meng and R. Blake, Neural bases of binocular rivalry, *Trends in Cognitive Sciences* **10**(11) (2006), 502–511.

[9] S. Veser, R.P. O'Shea, E. Schröger, N.J. Trujillo-Barreto and U. Roeber, Early correlates of visual awareness following orientation and colour rivalry, *Vision Research* **48** (2008), 2359–2369.

[10] J.C. Wilcke, R.P. O'Shea and R. Watts, Frontoparietal activity and its structural connectivity in binocular rivalry, *Brain Research* **1305** (2009), 96–107.
